# Mouse mammary tumour virus-like *env* nucleotide and p14 signal peptide are present in feline mammary carcinomas, but not in neoplastic or dysplastic canine mammary lesions

**DOI:** 10.1371/journal.pone.0200839

**Published:** 2018-07-24

**Authors:** Prospero Civita, Michele Menicagli, Claudia Scopelliti, Francesca Lessi, Francesca Millanta, Sara Borsacchi, Francesca Parisi, Giulia Freer, Mauro Pistello, Chiara Maria Mazzanti, Alessandro Poli

**Affiliations:** 1 Fondazione Pisana per la Scienza, Pisa, Italia; 2 Dipartimento di Scienze Veterinarie, Università di Pisa, Pisa, Italia; 3 Dipartimento di Ricerca Traslazionale e delle Nuove Tecnologie in Medicina e Chirurgia, Università di Pisa, Pisa, Italia; Baylor College of Medicine, UNITED STATES

## Abstract

Mouse mammary tumour virus-like (MMTV-like) is suspected to be involved in human breast cancer and it has been hypothesized that companion animals might have a role in viral transmission. The aim of our study was to investigate the presence of MMTV-like nucleotide sequences and viral protein in a larger number of feline (FMCs) and canine mammary carcinomas (CMCs) by nested PCR and immunohistochemistry. Results showed that the presence of MMTV-like *env* sequence in FMCs was 7% (6/86), while all the CMCs and canine dysplastic lesions scored negative. All PCR-positive FMCs scored positive for the MMTV p14 signal peptide of the envelope precursor protein of the virus. In contrast, all PCR-negative FMCs and canine mammary lesions were also negative for immunohistochemistry analysis. Canine and feline normal mammary gland tissues scored negative for both PCR and MMTV-p14 protein. Multiple nucleotide alignment of MMTV-like *env* gene sequences isolated from cat showed 97% and 99% similarity with HMTV and MMTV, respectively, while the others two presented some polimorphisms. Particularly the sequences of one of these two tumors showed a polymorphism (c.7575 A> G), that causes a previously unreported amino acid substitution (Thr > Ala). In conclusion, the results of our study showed the presence of MMTV-like sequences and viral protein in some FMCs. Further studies are needed to understand whether this virus does play a role in the development of FMCs, if MMTV-like is an exogenous virus as these data suggest and, in such a case, how and from whom this virus was acquired.

## Introduction

The mouse mammary tumor virus (MMTV) is the prototype of slow-transforming retroviruses and is now classified in the *Betaretroviridae*. This virus is known to cause mammary carcinoma and lymphoma in susceptible mice [1, 2]. MMTV can be transmitted either as an endogenous virus through the germ line or as an exogenous virus [3]. Endogenous MMTV is frequently detected in laboratory mouse strains, but most of these endogenous viruses do not produce viral infectious particles due to transcriptional regulatory or coding region mutation [4]. Horizontal transmission typically occurs from MMTV-infected dams to sucking offspring by MMTV virions present in the milk.

The involvement of a MMTV-like virus in human breast cancer has been proposed, based on the presence of MMTV-like sequences highly expresses in human breast cancer, and sharing over 95% identity with MMTV [5–8]. This hypothesis is also supported by the evidence that viral particles produced in primary cell cultures derived from human breast cancer are similar to those of MMTV [9].

Besides mouse and human, the association between MMTV or MMTIV-like virus has been demonstrated for other animal species. It has been observed that a MMTV variant isolated from mice can productively replicate both in canine and human cells by serial passages [10] and MMTV-like nucleotide sequences were detected in some feline and canine mammary tumors [11], but the role of MMTV-like virus in the induction of mammary tumors in these species has not been demonstrated. Furthermore, it has been hypothesized that companion animals might have a role in viral transmission of MMTV-like virus to humans, but no firm evidences have been accrued so far.

The aim of the present study was to investigate the presence of MMTV-like *env* nucleotide and protein sequences in feline and canine mammary dysplastic and neoplastic mammary lesions.

## Materials and methods

### Animals and tissue

#### Tissue sampling

All tissue samples, formalin fixed and paraffin embedded were retrieved from the archives of the Tumor Registry of the Department of Veterinary Science, University of Pisa, from January 2011 to December 2016. The samples were surgically obtained by mastectomy or nodule dissection from 77 bitches and 86 queens. Normal mammary gland tissues were collected during routine necropsies with the owners’ consent, from four bitches and six queens that died due to causes unrelated with mammary tumors.

Representative portions of each tumor were fixed in 10% neutral buffered formalin and routinely embedded in paraffin. Five-micrometer-thick-sections were stained with hematoxylin and eosin (HE) for histological evaluation; additional 5-μm sections were used for immunohistochemical studies. The mammary tumors were classified according to the World Health Organization classification [12] and tumors displaying different features were classified according to the most pronounced histological differentiation. Presence of lymphatic or stromal invasion was also recorded. Histological tumor grading was performed on HE-stained sections using a previously described classification for feline [13] and canine mammary tumors [14]. The mammary carcinomas were classified as well-differentiated carcinoma (WDC), moderately differentiated carcinoma (MDC), and poorly differentiated carcinoma (PDC).

#### Laser microdissection

A Zeiss automatic laser microdissection system (Laser Microdissection—PALM MicroBeam ZEISS Microscopy) was employed to select the epithelial cell population used in the study. Six-micron thick were cut from each paraffin block using a new microtome blade for each slide. Using the laser microdissection system a total of 200,000–300,000 μ m^2^ of epithelial cells were collected, stromal and inflammatory cells were carefully excluded. Because of the long experience of the laboratory with this method, selected areas picked with no difficulty [15].

### Molecular analyses

#### DNA extraction

Microdissected samples were kept overnight in lysis buffer containing proteinase K. Samples were processed for specific PCR amplification the next day and DNA was extracted from each microdissected area using Promega reagents according to the manufacturer’s protocol (Promega purification DNA FFPE). To avoid cross contamination, blank DNA samples (water) were processed in parallel with the tissue samples. The concentration of DNA was determined by dsDNA HS assay on Qubit 3 instrument (Invitrogen).

#### Detection of MMTV like env sequence by nested Fluorescence-PCR

Fluorescent-nested PCR was used to detect the presence of MMTV *env*-like sequence. Generated fluorescent amplicons were sized on an automatic DNA sequencer. The pairs of primer were designed on the basis of the sequence available in GenBank (accession no. AF243039) and designed in such a way to generate amplicons of 201 bp or less to ensure amplification of paraffin-embedded tissues. The outer primers yield a 201-bp fragment from nucleotide positions 231 to 430 of MMTV *env*-like, and the inner primers yield a 191-bp fragment (nucleotide positions 240 and 431). Sequences of the outer primers were: forward, 5 = -GATGGTATGAAGCAGGATGG-3 =; and reverse, 5 = -AAGGGTAAGTAACACAGGCA-3 =. Inner amplification was performed with the outer, reverse primer sequence of forward primers (5 = - AGCAGGATGGGTAGAACCTA-3 =).

Both PCRs were performed in 50 μ L containing 1x standard PCR buffer 1.5 mm MgCl2, 200 m each 2 = -deoxyribonucleoside 5 = -triphosphate, 0.5 nmol/L unlabeled reverse primer, 0.5 mol/L 6-FAM–labeled forward primer (Applied Biosystems, Milan, Italy), and 2.5 U Takara Ex Taq DNA polymerase. Input target template was 100-ng genomic DNA in the first-round PCR and 2 μl of first-round PCR product in the second round. The amplification profile was as follows: one cycle at 94°C for 10 minutes; 40 (first-round) and 30 (second-round) cycles at 94°C for 45 seconds, 58°C for 45 seconds, and 72°C for 60 seconds; and a final extension at 72°C for 7 minutes. To exclude PCR contamination, water controls and negative DNA samples were included every five samples in each run. As positive control DNA from MMTV-positive murine cell line Mm5Mt mammary carcinoma was used.

Fluorescent amplicons were analyzed by capillary electrophoresis and appeared as peaks in an electropherogram. The amplicon size was extrapolated from a molecular size ladder re-suspended in PCR buffer and run in parallel. Briefly, 3 μ l of PCR products from both amplification rounds were mixed with 0.5 μ l of ROX labeled size standard (Gene Scan 400 HD ROX; Applied Biosystems) and 11.5 μ l of formamide (Hi-Di Formamide; Applied Biosystems). After denaturation at 95°C for 3 minutes, samples were loaded onto an ABI PRISM 3100 automatic genetic analyzer and analyzed using GeneMapper software, version 3.1 (Applied Biosystems, Foster City, CA).

#### Research of murine DNA

The presence of contaminating mouse DNA was excluded by performing murine mitochondrial DNA and IAP LTRs PCR, according to Robinson et al. 2010 [16].

#### DNA sequencing

Sample that was positive to MMTV *env*-like was sequenced after clean up with the QIAquick PCR Purification Kit (Qiagen, Venlo, Netherlands) using Big Dye Terminator mix (Applied BioSystems, Warrington, UK). Sequencing reactions were run on an ABI3130 XL (Applied BioSystems, Warrington, UK).

#### Bioinformatics analysis: Multiple alignment and phylogenetic tree

Phylogenetic analysis of representative *Felis catus* retroviruses (FcERVs), used to root the tree, with previously identified MMTV sequences and gamma and beta retroviruses was performed to compare the sequences found herein and indicated as *ID* 86367 and *ID* 87768. FcERV gamma and beta families clustered with gamma retroviruses (X9927.1; X99929.2; X99925; X9934) and betaretroviruses (MPMV ; HERV_K) [17] in a phylogenic tree.

The nucleotide sequences were aligned using the CLC Sequence Viewer software for multiple alignment tool ClustalW package and neighbor-joining and UPGMA phylogenies package for phylogenetic tree and statistical analysis. The phylogenetic tree was inferred based on the 201-nucleotide sequence of the partial *env* gene of MMTV. The neighbor-joining phylogenetic tree [18] was constructed after 1,00 replicates.

### Pathological investigations

#### Immunohistochemistry

For immunohistochemistry we used an immunohistochemical staining method previously utilized to immunolocalize the MMTV-like p14 protein in human breast cancer [19] and validate by FISH analysis [20]. Four-micron thick sections were dewaxed in xylene and rehydrated through graded alcohols to water. Antigen retrieval was performed microwaving sections for 9 minutes in citrate/EDTA buffer (pH 7.8). Not specific peroxidase activity was blocked with 3% hydrogen peroxidase for 15 minutes, and non-specific binding prevented by incubation with normal goat serum for 10 minutes. Afterwards, incubation with 1:2000-diluted rabbit polyclonal antibody anti MMTV-p14 (kindly provided by Dr J Hochman, University of Jerusalrm, Israel) was performed for 2 hour at room temperature. Negative controls included the omission of the primary antibody. A biotin conjugated goat derived secondary antibody was applied followed by the enzyme-labeled streptavidin and substrate chromogen (Rabbit specific HRP/DAB-ABC detection IHC kit, ab 64261 abcam). Slides were counterstained with hematoxylin. Cytoplasmic/nuclear staining for MMTV-p14 were considered positivity.

## Results

### Pathological investigations

Canine breeds of the 77 subjects bearing mammary lesions sampled included mongrel (n = 11), German shepherd (n = 8), Pinscher (n = 6), Yorkshire terrier (n = 6), Brittany spaniel (n = 6), English Setter (n = 5), Boxer (n = 5), dachshund (n = 5), Beagle (n = 4), Labrador (n = 4), Jack Russel terrier (n = 3), Kurzhaar (n = 3), Pointer (n = 2), Spring Spaniel (n = 2), Cocker Spaniel (n = 2), Dalmatia (n = 2), Carlino (n = 2) and one Rhodesian ridgeback bitches. Of the 77 examined bitches 16 had mammary adenosis (mean age 9.7 years ± 2.4 years, range, 5–14 years), 8 benign simple adenomas (mean age 9.2 years ± 3.7 years, range 8–14 years) and 53 complex or simple carcinomas (mean age 10.4 years ± 2.5 years, range 6–15 years). The mean age of the four control bitches of different breeds was 8.5 years ± 1.3 years, range 7–10 years.

The feline breeds included European short hair (n = 78, 75 females and three males), Persian (n = five, all females), one Devon queen, one Maine Coon queen and one Siberian queens. All the subjects had a carcinoma and no benign lesions were detected. The mean age of examined cats was 10.5 ± 2.5 (range 7–14 years). The mean age of the six control queens was 8.6 ± 3.8 years.

Data about morphologic characteristics of canine and feline mammary malignant lesions are presented in Table 1. Canine malignant mammary tumours were classified as 10 grade complex carcinomas (8 WDCs and 2 MDCs) and 43 simple carcinomas: 18 tubulopapillary (9 WDCs, 7 MDCs and 2 PDCs), 23 solid (1 WDCs, 7 MDCs and 15 PDCs) and 2 PDC cribriform carcinomas. At the time of diagnosis lymphatic vessels around the tumour or lymph node invasion was detected in 28 carcinomas (52.8%; 8 tubulopapillary, 18 solid and 2 cribriform tumours), while the other 25 (47.2%) were locally infiltrative.

**Table 1:** Pathological features of the 53 canine and 86 feline mammary carcinomas examined.

Histologic classification		Canine				Feline			
		WDCs	MDCs	PDCs	Total	WDCs	MDCs	PDCs	Total
Complex		8	2		10				
Simple	Tubolopapillary	9	7	2	18	30	35		65
	Solid	1	7	15	23	2	8	10	20
	Cribriform			2	2			1	1

WDCs = Well differentiated carcinomas; MDCs = Moderately differentiated carcinomas; PDC Poorly differentiated carcinomas.

Feline mammary lesions were 86 feline simple mammary carcinomas: 65 tubulopapillary carcinomas (30 WDCs and 35 MDCs) 20 solid carcinomas (2 WDCs, 8 MDCs and 10 PDCs) and one cribriform PDC. Lymphatic or lymph node invasion was detected in 53 tumors (61.6%; 37 tubulopapillary, 15 solid and one cribriform tumours), while the other 33 (38.4%) were locally infiltrative.

### Laser microdissection

Laser microdissection system allows collecting only the selected epithelial components (neoplastic, dysplastic or normal mammary epithelial cells), when comparing the representative tissue sections before laser microdissection and after the use of microdissector.

### Detection of MMTV-like sequences and transcripts by nested-PCR

**Fig 1:**
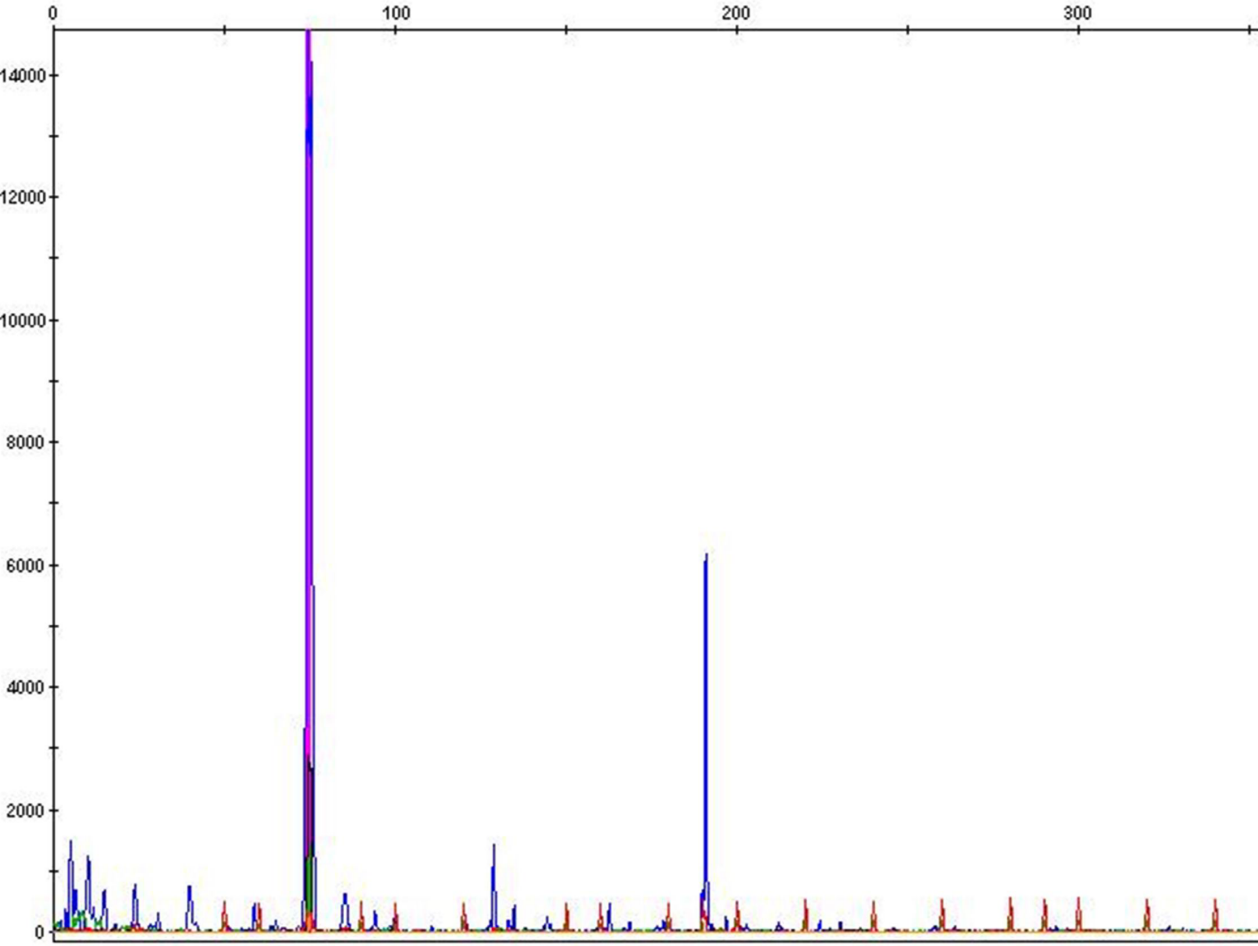
Fluorescent-PCR to detect MMTV-like sequence in feline mammary tumors. Fragment analysis show the blue peaks that represent the size of fragment (191bp) detected by capillary electrophoresis method.

The extracted DNA integrity and quality were validated by the PCR amplification of both cats and dogs GAPDH gene (data not shown). The results of PCR analysis performed to detect MMTV-like sequences in dysplastic and neoplastic tissue examined are presented in Fig 1.

Among the 86 feline mammary carcinomas examined, amplification of *env* sequences was observed in six tumors (7%). None the dysplastic and neoplastic canine mammary lesions were positive for MMTV-like *env* sequences as well as none of the canine and feline normal mammary tissues.

### MMTV (Env) protein immunolocalization

**Fig 2:**
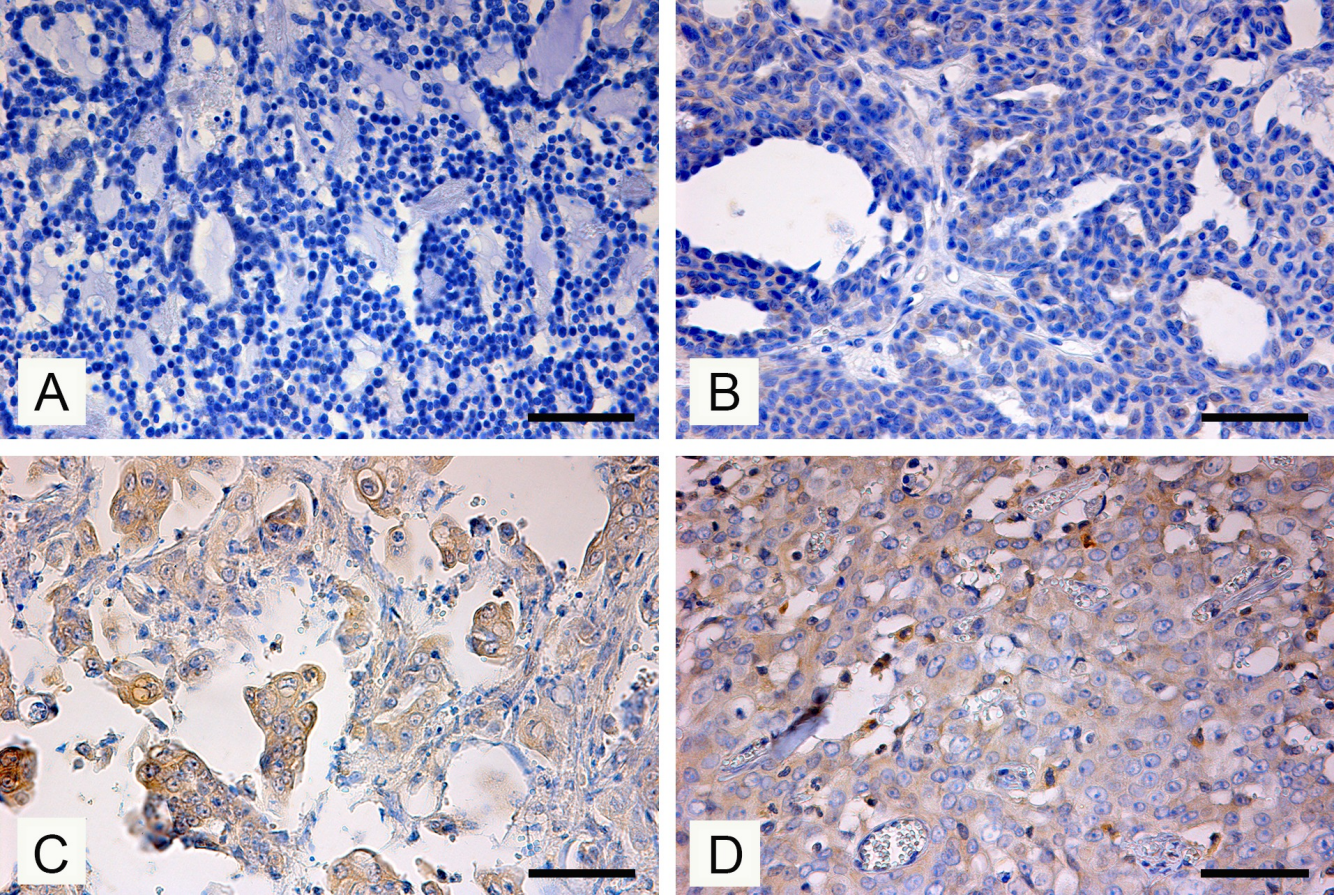
Immunohistochemical expression of p14 MMTV env protein in feline mammary carcinomas. (A) FMC # 84471. Tubulopapillary FMC PCR-negative and negative for MMTV p14 expression (Immunoperoxidase-DAB, Bar = 100 μm) (B) FMC # 84589. Tubulopapillary FMC PCR-positive, a weak cytoplasmic immunostaining was detected in some neoplastic epitrhelial cells (Immunoperoxidase-DAB, Bar = 100 μm). (C) FMC # 80613. Tubolopapillary FMC, some neoplastic epithelial cells show distict cytoplasmic MMTV p14 labelling (Immunoperoxidase-DAB, Bar = 100 μm). (D) FMC # 86367. Solid FM, a large number of carcinoma cells show cytoplasmic p14 staining, Scattered inflammatory cells associated with neoplastic proliferation resulted MMTV p14-positive (Immunoperoxidase-DAB, Bar = 100 μm).

MMTV-p14 is the signal peptide of the MMTV envelope precursor, localized in the nucleolus of the infected cells and involved in the nucleis cytoplasmic shuttling [21]. p14-staining expression was detected in the cytoplasm and nuclei of neoplastic mammary cells of feline mammary carcinoma PCR-positive for MMTV *env*-like sequences. The results of the immunohistochemical study are presented in Table 2. The p14-positive tumors were four tubulopapillary carcinomas (three locally infiltrative WDCs and one MDC with lymphatic invasion), one solid MDC carcinoma with lymphatic invasion and one locally infiltrative cribriform PDC carcinoma with lymphatic invasion. There was no correlation between FMCs grade and MMTV-like infection revealed by p14 immunohistochemistry. Feline and canine normal, dysplastic or neoplastic mammary tissues negative at the PCR analysis scored negative for the presence of viral protein (Fig 2A). Different staining pattern was detected in positive samples. These ranged from weakly staining (Fig 2B) to diffuse staining (Fig 2C). Some inflammatory cells present within the MMTV-like *env*-positive solid carcinoma showed a strong cytoplasmic staining (Fig 2D).

**Table 2:** Correlation between MMTV-like p14 expression and pathological findings of feline mammary carcinomas investigated.

Histologic classification		WDCs	MDCs	PDCs
Tubulopapillary		No. (%) of MMTV-like p14 positive cases	No. (%) of MMTV-like p14 positive cases	No. (%) of MMTV-like p14 positive cases
	Grade I n = 28	3/18 (16.7)	0/10 (0.0)	
	Grade II n = 37	0/12 (0.0)	1/25 (4.0)	
	Total N = 65	3/30 (10.0)	1/35 (2,9)	
Solid				
	Grade I n = 5	0/2 (0.0)	0/3 (0.0)	0/0 (0.0)
	Grade II n = 15		0/5 (0.0)	1/10 (10.0)
	Total n = 20	0/2 (0.0)	0/8 (0.0)	1/10 (10.0)
Cribriform				
	Grade II n = 1			1/1 (100.0)

Grade I FMCs = mammary tumors without lymphatic or lymph node invasion; Grade II = mammary tumors with lymphatic or lymph node invasion; WDCs = Well differentiated carcinomas; MDCs = Moderately differentiated carcinomas; PDCs Poorly differentiated carcinomas.

### Identification and analysis of MMTV-like sequences

The *env* sequences from two feline mammary tumors (indicated by their respective ID archival number) were aligned with NIH 3T3 (positive control for MMTV), mouse mammary tumor virus from HeJ mice, and HMTV (accession numbers AF228551.1 and AF243039) respectively.

**Fig 3:**
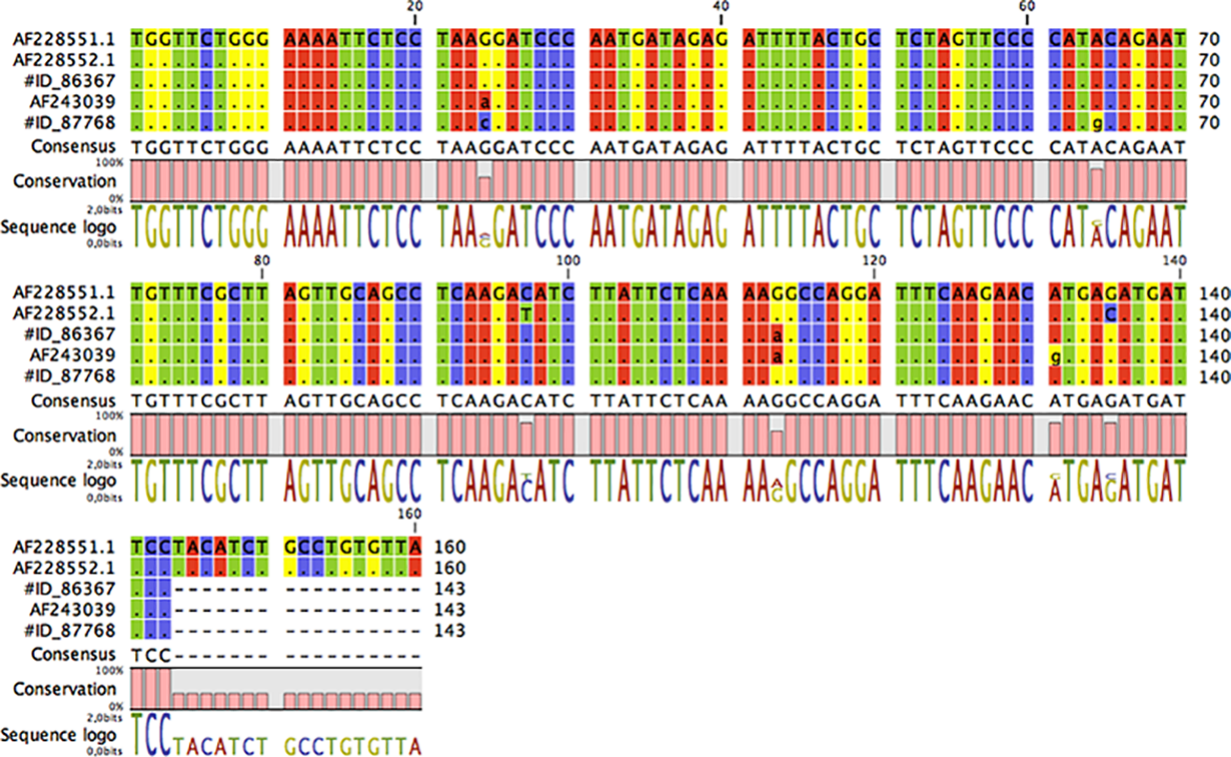
Multiple nucleotide alignment of the MMTV-like env gene sequences. A) The env sequences from two feline mammary tumors (indicated by their respective number) were aligned with NIH 3T3 (positive control for MMTV), mouse mammary tumor virus from HeJ mice, and HMTV (accession numbers AF228551.1 and AF243039, respectively). B) DNA sequencing, confirming the MMTV sequences. Surprisingly, one of them (Cat 87768) showed a polymorphism (c.7575 A> G), that cause aminoacyl substitution (Thr> Ala).

Results indicated sporadic nucleotide variations within the amplified *env* region (Fig 3). Overall our sequences shared homology for 97% and 99% for HMTV and MMTV, respectively.

Further, no significant similarity was found when our sequences were blasted or compared to the canine, feline and human genome sequences available in the GenBank database, indicating that amplified products did not belong to canine, feline, or human genomes or endogenous retroviruses.

### Phylogenetic analysis of MMTV-like sequences

**Fig 4:**
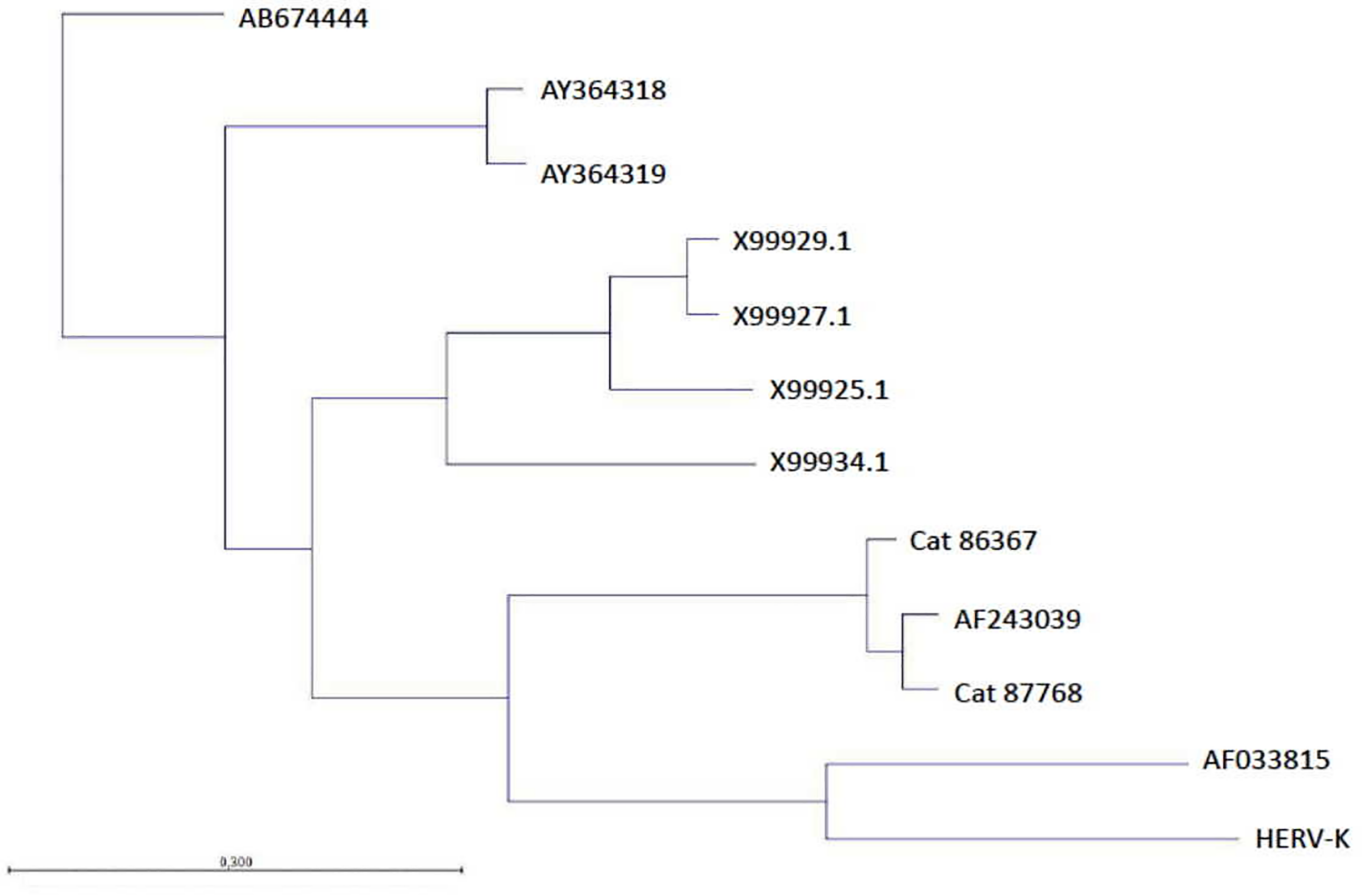
Determination of phylogentic relationships of Env sequences identified in different hosts. A neighbor-joining phylogenetic tree from nucleotide sequences of env of MMTV viruses. The tree was rooted to HERV-K. The robustness of individual nodes of the tree was determined by using bootstrap analyses of 1,00 replicates.

The neighbor-joining phylogenetic analysis showed that the MMTV detected from the examined cats were classified into different clusters compared MMTV detected in mice, humans and dog, indicating that the MMTV virus can be transmitted among these hosts (Fig 4). This inference was confirmed by rooting our sequences to *Felis catus* endogenous retroviruses (FcERV) database [17] and HERV-K database [22]. Rooting, also demonstrate that our sequences do not belong to FcERV and HERv-K family; the robustness of individual nodes and segregation was tested with bootstrap analyses of 1,00 replicates.

## Discussion

The possibility that a virus can be etiologically involved in sporadically occurred breast cancer in humans is consistent with a large body of scientific evidence accumulated since the mid-1990s and the hypothesis that animals could be implicated as mediators for viral transmission is particularly important. MMTV, belonging the *Betaretroviridae*, is the prototype of slow transforming retroviruses. MMTV has been definitively shown that is the cause of cause of mammary tumors in mice. The discovery of this virus prompted many investigators to explore a retroviral etiology also for breast cancer in humans [23]. In last years, amount of information about the possible existence of a human MTV has been accumulated. Viral particles and reverse transcriptase activity have been documented in milk and neoplastic tissues of women bearing breast cancer [24–26]. MMTV antigens have been detected in serum and tumors [27, 28] and exogenous MMTV-like sequences were amplified in 30% to 40% of human breast cancer [15, 29, 30]. These data and some epidemiological evidence [31, 32] suggest a viral etiology of breast cancer in women. Furthermore, two human genes encoding proteins highly related to the MMTV receptor (MMTVr) were identified [33] confirming the possibility that a MMTV-like virus can infect human cells.

Thus, the possibility of a zoonotic transmission of MMYV-like virus is an interesting hypothesis, supported by the xenotropic infection of non-murine cells and particularly cat kidney cells in particular culture conditions [34].

In this perspective, our retrospective study performed on a large number of FMCs succeeded to demonstrate the presence of MMTV-like sequences and MMTV p-14 antigen in mammary tumour in the feline species. These data confirmed and amplified previous studies performed by other authors [11] that demonstrated the presence of *env* and LTR MMTV-like sequences in 2/9 feline malignant tumors. In our study the percentage of MMTV-like positive FMCs was lower (7% vs 22.2%) and the presence of viral sequences was confirmed by the p-14 MMTV-like presence using an immunohistochemical staining method that localize the MTTV-like protein in the cytoplasm of epithelial exfoliated cells in human saliva, confirmed by FISH analysis that was capable of hybridizing also RNA sequences, supporting the presence of viral particles in the cytoplasm [20].

In addition, sequences highly similar (>95%) to the MMTV *env* gene amplified from human DNA, were also amplified from feline genomic DNA samples [21]. In the same animals sequence ~similar to the MMTV group antigen gene (gag) was amplified [21].

Our immunohistochemical study revealed that scattered inflammatory cells associated with neoplastic proliferation scored positive for the presence of p-14 MMTV-antigen. Retroviral particles characteristic of a MMTV-like virus have been detected in monocytes from patients with breast cancer [15] and MMTV-like sequences were identified in kitten thymus DNA confirming that lymphoid tissues can be infected by these MMTV-like retroviruses.

Many MMTV-like positive human breast cancer have similar histology to MMTV positive mouse mammary tumors and MMTV-like infection was frequently identified in benign breast neoplastic tissues [35]. It has been hypothesized that MMTV-like sequences represent, in humans, an event bound to the early steps of carcinogenesis [20] and the presence of MMTV-like sequences and antigen in tubulopapillary and WD and locally infiltrative FMCs seems to confirm the role of the virus in the early phase of mammary tumor development also in the feline species, even if an extensive study on pre-neoplastic lesions also in this species could give important confirmations.

Moreover, as phylogenetic relation of viruses from various host species provides evidence for predicting the transmission event, the phylogenetic relationship was further analyzed. Four MMTV-like *env* sequences showed 98% homology with the sequences of MMTV (AF243039), while the other two presented some polymorphisms. Particularly the sequences of the FMC # 87768 showed a polymorphism (c. 7575 A>G) that cause a previously unreported amino-acid substitution (Thr>Ala).

In our study none of the canine mammary lesions investigate scored positive for the presence of MMTV-like sequences and p-14 antigen. On the contrary, a previous study demonstrated the presence of MMTV-like *env* and LTR sequences in the 3% and 18.6% of 86 canine malignant tumour [11]. The PCR analysis used is very sensitive and therefore very unlikely that the failure to detect these sequences in the canine species is due to a methodological problem, although the lower number of malignant tumors or the possible geographic differences could explain these different results.

## Conclusions

Our study confirm the presence of MMTV-like sequences in feline mammary tumors and demonstrated for the first time the presence of p14-MMTV protein in neoplastic cells and inflammatory cells associated with the tumor. Further studies are needed to ascertain the presence of the entire proviral genome and understand whether this virus does play a role in the development of FMCs.
